# Disinfection byproducts formed during drinking water treatment reveal an export control point for dissolved organic matter in a subalpine headwater stream

**DOI:** 10.1016/j.wroa.2022.100144

**Published:** 2022-04-25

**Authors:** Laura T. Leonard, Gary F. Vanzin, Vanessa A. Garayburu-Caruso, Stephanie S. Lau, Curtis A. Beutler, Alexander W. Newman, William A. Mitch, James C. Stegen, Kenneth H. Williams, Jonathan O. Sharp

**Affiliations:** a Department of Civil and Environmental Engineering, Colorado School of Mines, Golden, CO 80401, United States; b Pacific Northwest National Laboratory, Richland, WA 99354, United States; c Stanford University, Stanford, CA 94305, United States; d Rocky Mountain Biological Laboratory, Gothic, CO 81224, United States; e Lawrence Berkeley National Laboratory, Berkeley, CA 94720, United States; f Hydrologic Science and Engineering Program, Colorado School of Mines, Golden, CO 80401, United States

**Keywords:** Climate change, Disinfection byproducts, Organic matter, Watershed, Water treatment, Water quality

## Abstract

Changes in climate, season, and vegetation can alter organic export from watersheds. While an accepted tradeoff to protect public health, disinfection processes during drinking water treatment can adversely react with organic compounds to form disinfection byproducts (DBPs). By extension, DBP monitoring can yield insights into hydrobiogeochemical dynamics within watersheds and their implications for water resource management. In this study, we analyzed temporal trends from a water treatment facility that sources water from Coal Creek in Crested Butte, Colorado. These trends revealed a long-term increase in haloacetic acid and trihalomethane formation over the period of 2005–2020. Disproportionate export of dissolved organic carbon and formation of DBPs that exceeded maximum contaminant levels were consistently recorded in association with late spring freshet. Synoptic sampling of the creek in 2020 and 2021 identified a biogeochemical hotspot for organic carbon export in the upper domain of the watershed that contained a prominent fulvic acid-like fluorescent signature. DBP formation potential analyses from this domain yielded similar ratios of regulated DBP classes to those formed at the drinking water facility. Spectrometric qualitative analyses of pre and post-reacted waters with hypochlorite indicated lignin-like and condensed hydrocarbon-like molecules were the major reactive chemical classes during chlorine-based disinfection. This study demonstrates how drinking water quality archives combined with synoptic sampling and targeted analyses can be used to identify and understand export control points for dissolved organic matter. This approach could be applied to identify and characterize analogous watersheds where seasonal or climate-associated organic matter export challenge water treatment disinfection and by extension inform watershed management and drinking water treatment.

## Highlights

• Drinking water records revealed DBP concentrations above health standards during spring freshet.
• Synoptic watershed sampling identified an export control point for organic matter.
• Lignin- and condensed hydrocarbon-like molecules were most reactive during chlorination.

## Graphical abstract

**Figure:**
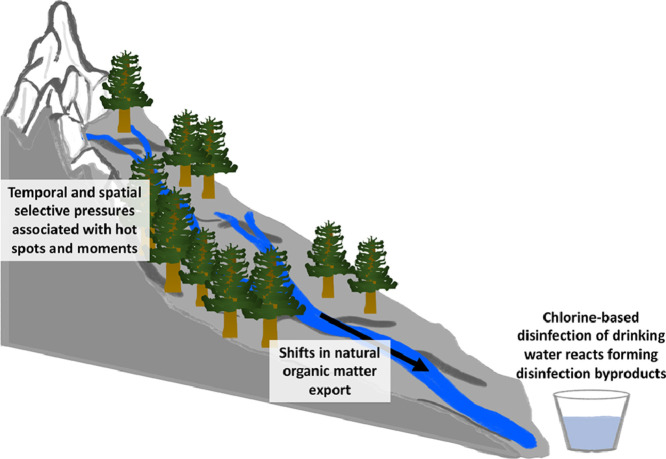

## 1 Introduction

Environmental fluctuations associated with weather, season, climate change, and acute disruptive events can have a pronounced influence on watershed biogeochemical processes. For instance, drought and insect-induced tree mortality can increase organic inputs to surface water sources and alter soil chemistry (Kramer and Chadwick, 2018; Mikkelson et al., 2013). Warmer temperatures can increase decomposition rates of available soil organic matter (OM) (Freeman et al., 2001), and soil freeze-thaw cycles have been associated with increased release by disrupting OM aggregates, plant roots, and lysing microbial cells (Tiwari et al., 2019). During precipitation events, these variables can lead to a higher flux of aqueous OM (Ågren et al., 2010) which can impact downstream water quality. With an estimated 74% of water used in the United States sourced from surface waters, biogeochemical shifts could have large-scale impacts on downstream communities (Dieter et al., 2018).

Water professionals are faced with the task of adapting treatment processes as source water chemistry and regulatory requirements change. Higher concentrations of OM specifically pose challenges during chemical disinfection which is used at 70% of municipal treatment facilities within the United States (AWWA, 2018). While an efficient method to inactivate pathogens, ancillary reactions with aqueous OM can lead to the creation of carcinogenic disinfection byproducts (DBPs) (Richardson and Plewa, 2020; Villanueva et al., 2015). More than 700 DBPs have been identified, and eleven (four trihalomethanes, five haloacetic acids, bromate, and chlorite) are currently regulated in the United States (Richardson and Plewa, 2020). With the Disinfectants and Disinfection Byproducts Rules enacted by the EPA in 1998 and 2006, water systems adding disinfectant during treatment must comply with the established maximum contaminant levels (MCL). In addition, distribution systems serving a population greater than 500 must conduct quarterly sampling for total trihalomethanes (TTHM) and haloacetic acids (HAA5) to achieve regulatory compliance (U.S. EPA, 2010).

The presence of DBPs is a prominent concern for municipal treatment facilities (AWWA, 2020), but formation can be mitigated through a variety of approaches that target additional removal of dissolved organic carbon or with the adoption of alternative disinfection processes. While prior studies have focused on DBP reduction by changing conventional unit processes during drinking water treatment (Andersson et al., 2020; Postigo et al., 2021; Zhang et al., 2021), those methods present an additional economic and infrastructure burden that can be particularly pronounced for smaller facilities. In addition, disinfection methods that use alternatives to chlorine can form other, less understood byproducts (Richardson and Plewa, 2020). As OM is a complex constituent by nature, screening typically focuses on total organic carbon concentrations, normalization to specific UV absorbance as an indicator of reactivity, and oxygen demand as an indicator of bioavailable organics (U.S. EPA, 2005). These approaches however do not capture structural properties of OM, which are important indicators for DBP formation and can vary across watershed landscapes (Yamashita et al., 2011). Further, these approaches do not capture contributions from specific structural properties that could mechanistically inform mitigation strategies for DBP formation during water treatment and proactive management of watershed source zones.

In this study, we focused on the Coal Creek subalpine watershed in Colorado to find localities within the landscape and temporal periods that contribute disproportionately toward higher aqueous OM flux and DBPs formed during drinking water treatment. The terms biogeochemical “hot spots” and “hot moments” have been used in association with disproportionality high and localized export over short periods, respectively, to understand the episodic release of mobilized OM (McClain et al., 2003). These concepts have evolved to more succinctly describe the spatiotemporal release of organics as “export control points”, which are defined as areas where high rates of reactants are exported into receiving streams during hydrologic events (Bernhardt et al., 2017). We hypothesized that an export control point for OM could be identified within this small (53 km^2^) headwater mountain catchment. We further theorized that this understanding could be used to reveal reactive chemical classes that link to DBP formation during drinking water treatment. To address these research goals, we used historical records of drinking water DBP monitoring to assess drivers of a hot moment combined with synoptic stream sampling to identify the source (hot spot) and quality of OM release. Dissolved OM (DOM) in the waters sourced from this export control point was further characterized to better understand specific properties associated with DBP formation.

## 2 Methods and materials

### 2.1 Watershed attributes & sampling

The Coal Creek watershed is the primary water source for the town of Crested Butte, CO and serves a population of approximately 2500 (CDPHE, 2021a). The total drainage is 53 km^2^ with several tributaries that descend a variety of land covers (Zhi et al., 2019). The total watershed land cover consists of 64% evergreen forest, 20% herbaceous vegetation, 9% deciduous forest, 4% barren land, 2% woody wetland, and 1% developed open space (NLCD, 2011). The southern section of the basin is heavily forested with conifer trees under increased stress from bark beetle impact starting in 2015 (CSFS, 2016). The northern reach contains three abandoned mines and an iron fen associated with legacy metal export (Ryan et al., 2007). The climate of the area is characterized by a nearby snow telemetry (SNOTEL) station on Mt. Crested Butte (38.89° N, 106.95° W, elevation 3100 m). The average total precipitation from 1990 to 2010 was 80 ± 15 cm snow water equivalent (SWE) with more than half (48 ± 14 cm SWE) falling as snow (Table S1). Snow cover is dominant typically November - June. The past five years have been variable with low moisture conditions in 2018 quantified by below-average total precipitation and above-average summer days followed by a return to average conditions in 2019 and severe drought conditions in 2020 and 2021 (Table S1).

Coal Creek begins near Lake Irwin (IR-00) from a portion of the lake diverted as a trans-basin diversion. Drinking water is diverted from the creek approximately 10.5 km downstream from Lake Irwin near site Coal-11 (Vaughter, 2013). Hence, this sampling location served as the most representative proxy for influent water before treatment (Fig. S1). The watershed has established sampling locations from prior studies (Blazewicz et al., 2012; CCWC, 2018; Vaughter, 2017). Three locations approximately equidistant from the lake to the water diversion (CC-5, Coal-20, and Coal-11) were selected for targeted analyses in 2020 and 2021 that integrated FTICR-MS and DBP-FP analyses as described below. These targeted sampling events were conducted in July and October 2020 to align with routine quarter 3 and quarter 4 monitoring by the drinking water facility and June 2021 to align with synoptic sampling (Table S2). Higher resolution synoptic sampling was conducted two days after peak flow on June 8th, 2021 and during falling limb flow on July 28th, 2021 to better constrain the hot spot region. The samples were collected every 1 km or less along the transect beginning at the Lake Irwin outlet and ending at the lower end of the transect with major tributaries included. All synoptic samples were analyzed for UV absorbance at 254 nm (UV_254_) with a subset receiving more comprehensive analyses (Table S2). Samples were collected and shipped on ice from Crested Butte to the Colorado School of Mines for processing. Analyses included dissolved organic carbon (DOC), UV_254_, specific UV absorbance (SUVA), and fluorescence excitation-emission matrix spectra (EEM) conducted at the Colorado School of Mines. In addition, a subset of the bulk samples was chlorinated for DBP formation potential tests (DBP-FP) and measured at Stanford University while Fourier-transform ion cyclotron resonance mass spectrometry (FTICR-MS) analysis was performed at the Pacific Northwest National Laboratory (PNNL) to compare pre and post chlorinated samples. Each method is described further in the following sections.

### 2.2 Organic matter and FTICR-MS analyses

The 2020–2021 creek sample collections were shipped on ice to the Colorado School of Mines for processing. A fraction was filtered at 0.45 µm and acidified with hydrochloric acid for DOC analysis using a Shimadzu TOC-550A Total Organic Carbon Analyzer. In addition, unacidified 0.45 µm filtrate was analyzed for UV_254_ using a DU 800 Spectrophotometer. SUVA was calculated by normalizing the UV_254_ values with the respective DOC concentrations in mg/L following Environmental Protection Agency Method 415.3 (Potter and Wimsatt, 2009).

Unacidified filtrate was also used for EEM analysis using a Horiba Aqualog 3D Fluorometer and the Aqualog 3.6 software. Experimental settings were conducted at an integration time of 1 s, excitation increments of 3 nm, and emission increments of 2.33 pixels. Samples were corrected for the inner-filter effect using the Aqualog software. The samples were blank corrected and normalized to Raman units. Processed sample data was further analyzed using R Studio version 3.5.2 (R Core Team, 2020) and R package eemR to determine the biological (Bix), fluorescence (Fi), and humification (Hix) indices calculated per the methods of Massicotte (2019).

FTICR-MS analysis was conducted on a subset of the pre and post chlorinated samples (formation potential as described in the next section). The samples were prepped for FTICR-MS analysis at the Colorado School of Mines by filtering both the raw (Pre-chlorinated) and reacted (Post-chlorinated) samples with a 0.22 µm polyethersulfone membrane filter (Millipore Sterivex, USA) into amber vials pre-acidified with 85% phosphoric acid. The samples were stored at 4 °C and shipped overnight on ice to PNNL. Once received, the samples were normalized to a final DOC concentration of 1.5 mg C L^−1^, acidified to pH 2 with 85% phosphoric acid, and extracted using PPL cartridges (Bond Elut), following Dittmar et al. (2008). FTICR-MS data was gathered following the methods described by Garayburu-Caruso et al. (2020). In brief, a 12 Tesla Bruker SolariX Fourier transform ion cyclotron mass spectrometer (Bruker, SolariX, Billerica, MA, USA) located at the Environmental Molecular Sciences Laboratory in Richland, WA was used to collect mass spectra of the pre- and post- chlorinated transect samples. The FTICR-MS was equipped with a standard electrospray ionization (ESI) source and data were acquired in negative mode with an ion accumulation time of 0.08 and 0.1. Peaks were aligned (0.5 ppm threshold) and chemical formulas were assigned using Formularity (Tolić et al., 2017). The Compound Identification Algorithm in Formularity considered the presence of C, H, O, N, S, and P and excluded other elements. It is important to note that FTICR-MS provides a non-targeted approach to reliably identify molecular formulas of organic molecules with masses between 200 and 900 *m/z*. However, it is not quantitative and does not provide information about the structure of the molecular formulas identified. Final data analysis and plots were conducted in R Studio version 3.5.2 following the methods outlined in Garayburu-Caruso et al., 2020. The R package ftmsRanalysis (Bramer et al., 2020) was used to remove peaks outside of the confidence range of 200–900 *m/z*, calculate molecular formula properties, assign chemical classes using O:C and H:C ratios (i.e., Van Krevelen classes), and generate Van Krevelen plots (Kim et al., 2003). Variation in the contributions of different chemical classes was quantified for each sample as the number of unique formulas in each chemical class divided by the total number of unique formulas. We refer to the resulting fraction for each chemical class in each sample as chemical class 'relative counts.' Significant differences between the pre- and post-chlorinated samples were compared using the Mann Whitney Wilcoxon test.

### 2.3 Disinfection byproduct (DBP) formation potential (FP) tests

The remaining bulk samples were filtered at 0.45 µm and prepared for DBP-FP analysis according to standard method 5710 to quantify the absolute potential for DBP formation (APHA, 2017). Chlorine demand tests determined the dose of sodium hypochlorite that would achieve a residual chlorine concentration of 3–5 mg/L for optimal FP of each sample during a seven-day reaction period (APHA, 2017). In brief, reagent grade 4.00–4.99% sodium hypochlorite and deionized water were used to prepare a stock hypochlorite solution. A diphosphate buffer (1 M, pH 7) and ascorbic acid (25 g/L) quenching solution were prepared. Amber vials were used for all reactions and pretreated with sodium hypochlorite stock solution filled headspace free for 24 h (U.S. EPA, 2019). Based on the DOC results for each sample, chlorine dose volumes were calculated to achieve chlorine:DOC mass ratios of 1, 3, 5, 7, and 10. Each filtered sample was added to 5 × 40 mL vials and buffered to 10 mM. The determined hypochlorite stock solution volume was dosed into one of the five vials for each ratio. Headspace-free vials were reacted in the dark for seven days at room temperature after which the residual chlorine concentration was measured using a Hach Chlorine Free & Total colorimetric test kit to determine which chlorine:DOC mass ratio achieved a residual chlorine concentration of 3–5 mg/L.

After this dose was determined, bulk samples were filtered in 2 × 250 mL amber bottles, buffered, dosed with hypochlorite stock solution, and filled headspace free. After a seven-day reaction time in the dark, one of the 250 mL reacted vials for each sample was filtered at 0.22 µm and preserved with 85% phosphoric acid for FTICR-MS analysis as described in Section 2.2. The second reacted 250 mL vial was quenched to achieve a concentration of 33 mg/L ascorbic acid and acidified with 5% v/v sulfuric acid to reach a pH of 3.7. The DBP samples were stored headspace free at 4 °C and shipped overnight on ice to Stanford University where the DBPs were extracted according to EPA Methods 551.1 (U.S. EPA, 1995) and 552.3 (U.S. EPA, 2003) and analyzed using gas chromatography-mass spectrometry as described previously (Chuang et al., 2019). A suite of known DBP compounds was analyzed including THMs (chloroform, bromodichloromethane, dibromochloromethane, bromoform), the 5 regulated HAAs (dibromoacetic acid, dichloroacetic acid, bromoacetic acid, chloroacetic acid, trichloroacetic acid), additional HAAs (bromochloroacetic acid, bromodichloroacetic acid, chlorodibromoacetic acid, idoacetic acid, tribromoacetic acid), haloacetonitriles, haloacetaldehydes, iodinated trihalomethanes, chloropicrin, and haloketones.

### 2.4 Historical data access & analysis

Historical DBP data were analyzed from 2005 to 2020 to understand seasonal and decadal trends at the water treatment facility of Crested Butte. The DBP data is regularly reported by the Crested Butte drinking water treatment facility and was obtained from the Colorado Department of Public Health and Environment (CDPHE) drinking water compliance database (CDPHE, 2021a). DBP concentrations were available at annual quarterly intervals for total trihalomethanes (TTHM) and the five regulated haloacetic acids (HAA5) in addition to the individual compounds that makes up each group. Linear trend lines for TTHM and HAA5 were fitted with the raw data using the ordinary least square regression fits in Tableau version 2020.4. To account for seasonality, the data were additionally separated by annual quarterly reporting dates for linear trend line analysis across each quarter. *P* values less than 0.05 were considered significant.

Historical Coal Creek daily hydrograph data was obtained from the U.S. Geological Survey (USGS) database for site number 09111250: Coal Creek ABV McCormick Ditch (38.872 ° N, 106.985 ° W, elevation 2700 m). The hydrograph data was available starting in October 2014, which allowed for comparative analyses with the historical DBP data from 2015 to 2020. Historic DOC concentrations near the drinking water diversion at Coal-11 (Fig. S1) from 2016 to 2020 have been previously published (Zhi et al., 2019). Time series using the 2015–2020 flow rates and 2016–2020 DOC concentrations were plotted in R Studio version 3.5.2. Pearson correlations were determined between the historical DBP concentrations and DOC as well as DBPs and flow in R Studio and plotted in Tableau version 2020.4. A *P* value less than 0.05 was considered significant for all linear fits and correlations. Finally, daily DOC mass loadings were determined with the historical hydrograph and DOC data by multiplying the DOC concentrations in mg/L by flow in m^3^/s with each associated date and appropriate unit conversions to return units of kg/day. These daily calculations were used to determine monthly averages for each year.

## 3 Results and discussion

### 3.1 Seasonal and decadal trends in DBPs during drinking water treatment

**Fig. 1:**
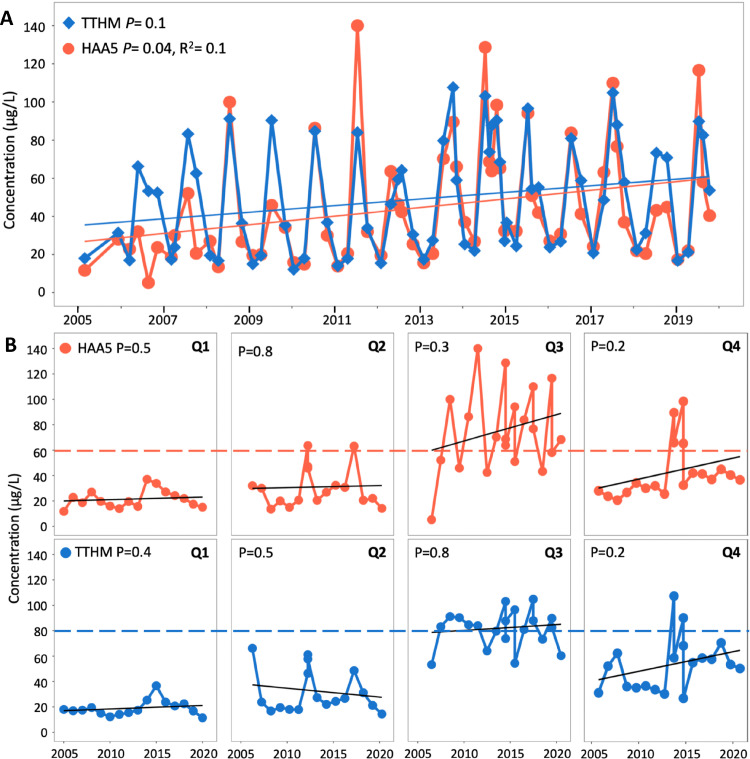
Long-term trends of total trihalomethanes (TTHM) and haloacetic acids (HAA5) from the Crested Butte treatment facility over the past fifteen years. (A) TTHM and HAA5 water quality report data are shown from 2005 to 2020 and broken down by (B) quarterly trends with respective maximum contaminant levels in dotted lines. Linear trendlines and the respective *P* values indicate linear fit significance. R^2^ values are shown only for significant trends.

Disinfection byproducts form when chlorine or other oxidants react with organic matter in water. As a result, trends in DBP concentrations can reflect carbon mobilization in the source water with implications for community health (Brouillard et al., 2016; Mikkelson et al., 2013). The historical DBP concentrations from the Crested Butte treatment facility served to discern trends of source water reactivity with chlorine-based disinfection. While the temporal resolution of this dataset was confined to four samples per year, seasonal trends of HAA5 and TTHM concentrations from 2005 to 2020 revealed repeated annual peaks in DBP concentrations after spring snowmelt (Fig. 1A). Over the past fifteen years, the observed peak values frequently exceeded the maximum contaminant levels (MCLs) of 80 and 60 μg /L for TTHM and HAA5, respectively. Interestingly, these higher concentrations were regularly associated with the third quarter of annual sampling (Q3) in July (Fig. 1B). While the Crested Butte drinking water treatment facility is in regulatory compliance with annual averages below MCLs (U.S. EPA, 2010), the trends and concentrations are alarming. Of the individual byproducts measured in the past five years, the regulated total HAA5s were dominated by dichloroacetic (42 ± 3%) and trichloroacetic (55 ± 4%) acids while TTHMs were dominated by chloroform (85 ± 9%) (Fig. S2).

Based on the long-term trends of HAA5 and TTHM concentrations at the treatment facility, it appears that Coal Creek hosts a seasonal and reproducible hot moment for DBP formation trending toward higher future concentrations (Fig. 1). Specifically, fitted linear trends using ordinary least squares regression returned significant increases (*P* = 0.04, R^2^=0.1) in HAA5 concentrations; however, an analogous increase in TTHM lacked this significance threshold (*P* = 0.1). Quarterly trends further revealed that Q3 (typically sampled in July) and to some degree Q4 (typically sampled in October) are driving the temporal increases (Fig. 1B). These trends could be associated with watershed stress associated with climate change, supported by previous studies in analogous forested ecosystems that experienced heightened tree mortality that correlated with increased DBPs at drinking water facilities (Brouillard et al., 2016; Mikkelson et al., 2013). In addition, TTHM and HAA5 formation could be linked with the ambient temperature of the environment, as demonstrated in a study that modeled future climate-related trends in DBPs and revealed a 1.8 °C increase in mean summer temperatures could lead to a 39% increase in TTHMs in Scotland (Valdivia-Garcia et al., 2019). We were unable to directly associate the water treatment facility DBP concentrations with influent organic carbon or temperature in this study. However, the Crested Butte region has experienced recent shifts in fewer total days of minimum air temperatures less than 0 °C and above average total maximum air temperatures greater than 20 °C (Table S1).

### 3.2 Hydrochemical associations

**Fig. 2:**
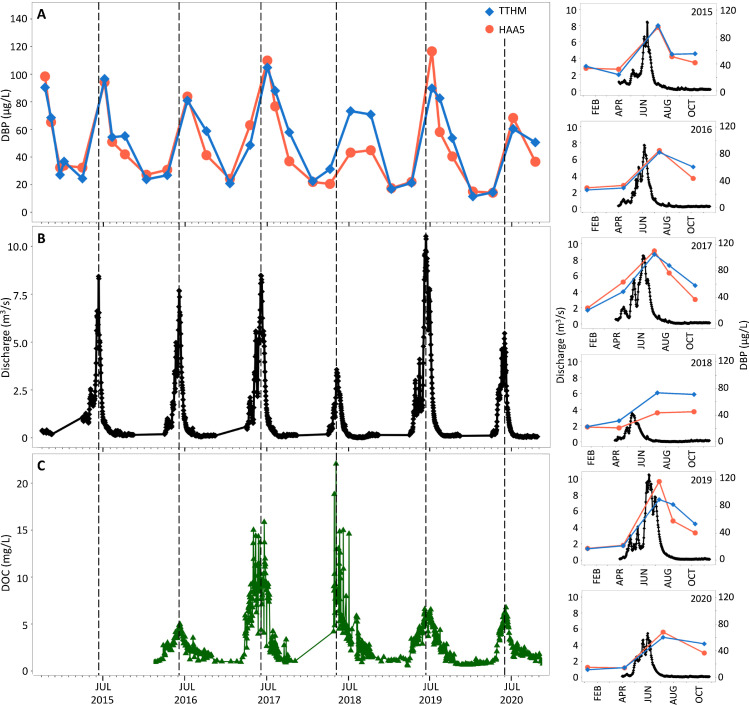
Seasonal trends in DBP concentrations after drinking water treatment are associated with peak flow and increases in DOC. (A) The TTHM and HAA5 concentrations from the water treatment facility are plotted with the respective (B) Coal Creek hydrograph and (C) Coal-11 DOC concentrations. Dashed lines indicate the annual peak flow. The right frame highlights the offset of peak flow with the timing of water quality monitoring for each year.

The quantity and structure of natural OM present in source waters are also precursors to DBP formation during treatment. As a result, the historical hydrograph and Coal-11 DOC concentrations were used for targeted analysis to understand the relationships between stream hydrochemistry and DBP concentrations after treatment. Over the past five years, the reported TTHM and HAA5 concentrations have consistently exceeded the EPA MCLs during Q3 sampling. While low in temporal resolution, these seasonal peaks in DBPs overlap with peak flow and DOC concentrations (Fig. 2). In the past five years peak flow has occurred near June 1st ± 13 days at 7 ± 3 m^3^/s while peak DOC has occurred near June 5th ± 16 days at 11 ± 7 mg/L. By extension, HAA5 concentrations correlated with both stream DOC (*P* = 0.001) and flowrates (*P* = 0.02) while TTHM concentrations correlated with DOC (*P* = 0.03) (Fig. S3). Consequently, this hot moment for DBP formation in Crested Butte extends to DOC mobilization and flow in association with the Q3 season of increased seasonal temperatures and snowmelt.

This observed temporal window is relatively brief but responsible for the majority of DOC export from the watershed each year with an average 78 ± 10% of DOC mobilized in May- June from 2015 to 2020. This aligns with a previous study across 30 forested watersheds wherein up to 86% of total DOC was exported during seasonal events associated with increased discharge and water temperatures (Raymond and Saiers, 2010). It is noted that the hydrograph data in this present study ends in October due to regularly frozen surface conditions that persist from November-May (Fig. S4). Despite these missing data, comparatively low DOC concentrations were observed during the colder months of November-March of 2016–2020 in contrast to the warmer months of April-October. By extension, we assumed lower DOC mass loading occurs during the winter months as a result of lower flow and DOC. This assumption is supported by a recent study that concluded higher temperatures are a major driver of DOC concentrations with release influenced by high flow during wet conditions (Wen et al., 2020).

The interplay between seasonal drivers of DOC export and temperature on reaction rates suggests that climate shifts could have a significant role in DBP formation. Specifically, enhanced DOC export has been associated with event-controlling snowmelt and storm events across a variety of watersheds including mountain and forested catchments (McKnight et al., 2001; Raymond and Saiers, 2010), high-latitude watersheds (Finlay et al., 2006), and agriculture-dominated watersheds (Qiao et al., 2017). DOC concentrations and lability (e.g., sugar and protein content easily consumed by microbes) have been shown to positively correlate with frost duration in upper soil horizons of riparian zones exposed to frost (Haei et al., 2011). In addition, peatland studies have associated increased DOC and humic acid-like organics with more frost-free days (Eskelinen et al., 2016) and higher temperatures (Fenner et al., 2007). These findings provide a possible explanation for the increasing trend in DBP concentrations over the past fifteen years that inversely mirror significant decreasing trends in total ice and frost days in Crested Butte (Fig. S5) and could contribute to higher DOC availability for release during spring snowmelt. Hence, cumulative climate shifts can impact downstream DOC concentrations and reactivity during drinking water treatment. However, DBP formation is also dependent on the treatment processes, which can change with time (Andersson et al., 2019).

Specific to this study, the Crested Butte water treatment facility involves a relatively simple system with few publicly reported modifications, none of which include changes to the chlorination process (CDPHE, 2021b). A holding pond provides storage for surface water flows. Coal Creek is the primary water source for the town and a secondary intake from Wildcat Creek (Fig. S1) is used for emergencies (Stantec, 2005). From the holding pond, incoming water is dosed with potassium permanganate to mitigate metal contamination from legacy mining operations in the watershed. This pretreatment is followed by microfiltration and sodium hydroxide dosing for pH and alkalinity adjustments. Calcium hypochlorite is added for pathogen disinfection and flows through clear wells and storage tanks to achieve a targeted disinfection contact time. This likely is also the stage most directly involved in the formation of disinfection byproducts when chlorine reacts with the remaining DOC in the system. Broader studies have assessed a variety of water treatment facilities for associated processes that can impact DBP formation. However a recent study determined despite differences in treatment across water distribution systems in Scotland, water conditions were significant predictors with the ambient temperature of the environment as the primary determinant of TTHM and HAA5 and concentrations of DOC as a secondary determinant (Valdivia-Garcia et al., 2016). Additionally, treatment-related variables of reaction time and pH have a significant influence on DBP formation (Doederer et al., 2014). Water treatment potentially represents a confounding variable in observed DBP trends, therefore synoptic and discrete sampling was conducted along the Coal Creek transect to better understand water properties before treatment.

### 3.3 Synoptic sampling to identify regions of enhanced export

**Fig. 3:**
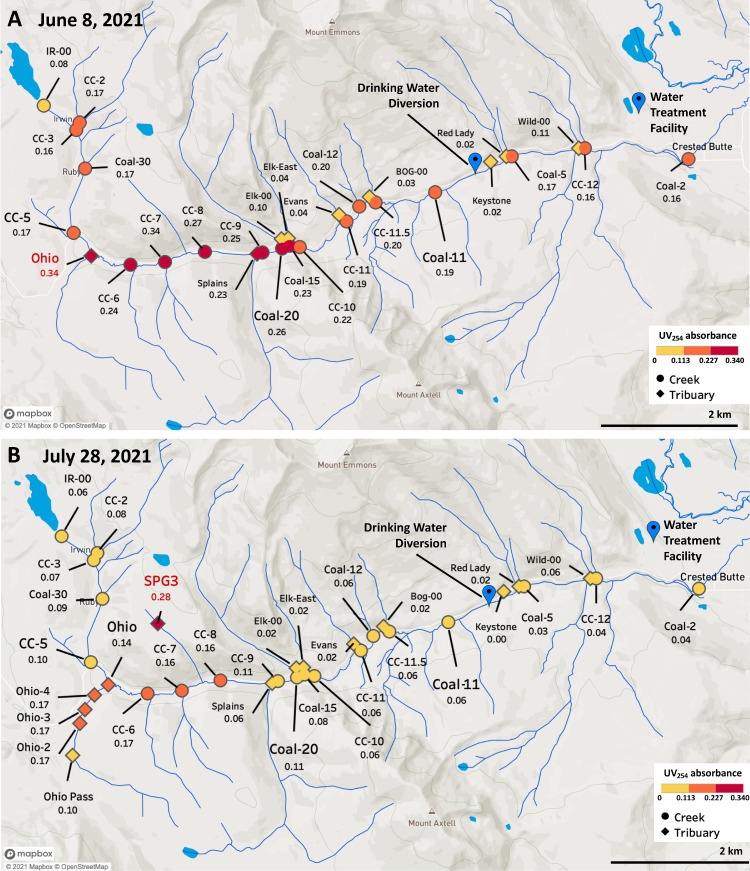
Synoptic sampling of the transect in 2021 revealed a hot spot for DOC export downstream of the Ohio fork. (A) UV_254_ absorbance values from June 2021 and (B) July 2021 are shown. Full DOC and UV_254_ values are provided in Tables S3 and S4. The base map was created in Mapbox (OpenStreetMap contributors, 2021) with points plotted in Tableau (Tableau Software, 2021).

To evaluate whether a particular region was disproportionately responsible for reactive organic export, a synoptic sampling campaign was conducted two days after peak flow on June 8th, 2021. Synoptic sampling focused on UV_254_ and DOC concentrations, which are strong indicators of HAA5 and TTHM formation potential (Brouillard et al., 2016; Golea et al., 2017). Correlations of these indicators with DBP formation potential in Coal Creek are investigated further in the next section. As depicted in Fig. 3, indicator values peaked in the upper reach of the Coal Creek transect near the Ohio fork in proximity to an extensive riparian wetland area with beaver activity as evidenced by dams. Indicator values began to decrease after this point (Fig. 3A, Table S3). Samples from downstream tributaries returned comparatively low values suggesting that dilution possibly in combination with transformation processes were responsible for this decrease. A second synoptic sampling campaign was conducted on July 28th, 2021 at lower flows and DOC concentrations to target tributaries within the identified higher export region. Similar to the peak flow transect, UV_254_ measurements returned higher values along the Ohio fork (red text in Fig. 3A; orange points in Fig. 3B) from the southern section of the basin and downstream to CC-8. In addition, a tributary sourced from a peat-like wetland in the northern region of the basin (SPG3, red point and text in Fig. 3B) returned the highest UV_254_ values observed in our study. This suggests that drainages on both sides of this reach may be contributing to organic loading (Fig. 3B, Table S4). Flow measurements conducted during the falling limb in 2021 upstream of the fork at Coal-30 (0.014 m^3^/s) and downstream at CC-6 (0.031 m^3^/s) revealed that the Ohio fork provides approximately 30–50% of headwater flow to Coal Creek, with a margin of error depending on season, the extent of water diversions from Lake Irwin to the total discharge, and groundwater flow. The northern SPG3 tributary consisted of lower flow that was too modest for flowmeter measurements.

The identification of this export control region for organic matter sets a foundation for a more detailed inquiry into ecosystem contributions that are beyond the scope of our current study. Land cover is an important variable in understanding watershed inputs as aboveground litter can influence DBP formation and different plant litter leachates can produce comparatively different DBP profiles (Franklin et al., 2021). The Ohio fork is sourced from the southwest section of the watershed and is primarily composed of spruce-fir forests that have experienced a degree of spruce beetle impact. This is in contrast to the tributaries draining from the north side which are more dominated by grasslands with peat-like wetlands near SPG3 (Alexander and Brown, 2009; NLCD, 2011). Disruptive events such as insect-induced tree mortality and fires have been associated with changes to soil DOC and increased DBP formation downstream (Brouillard et al., 2016; Mikkelson et al., 2013; Olivares et al., 2021; Uzun et al., 2020). Peatlands have been associated with higher DOC and aromaticity during summer rewetting events, and past studies that have explored catchments dominated by forests and wetlands revealed a link between late-season drought and increased DOC concentrations (Chen et al., 2020; Tiwari et al., 2019). Snowmelt dynamics add further complexity where elevation and slope can impact runoff and soil moisture (Ryan et al., 2007), and the timing of snowmelt across local climates can impact porewater DOC in association with litter decomposition (Leonard et al., 2021).

### 3.4 Formation potential of disinfection byproducts in Coal Creek

We expanded our inquiry to connect this ecosystem control point to dissolved organic carbon (DOC) reactivity. Correlative analyses of TTHM and HAA5 formation potential from the creek samples revealed significant relationships with associated DOC and UV_254_ values (Fig. S6, Table S5). While these measurements are regularly used in water treatment plant monitoring, this lends credence to the application of comparatively rapid and cost-effective screening using UV_254_ during synoptic watershed sampling as depicted in Fig. 3. Specific UV absorbance (SUVA) significantly correlated with DBP-FP as well, but with lower R^2^ values in contrast to DOC and UV_254_. This is consistent with prior investigations in Colorado forested watersheds and treatment facilities (Beauchamp et al., 2018; Brouillard et al., 2016). The upstream peak DBP-FP concentrations quantified in this study in June 2021 (Tables S3, S4) have implications for understanding total DBP during drinking water treatment when combined with flow and DOC monitoring as depicted in Fig. 2. Specifically, annual sampling conducted by the Crested Butte treatment facility in early July was almost one month after peak DOC (June 5th ± 16 days); hence, this quarterly sampling benchmark in early July misses the true peak in DBP concentrations.

**Fig. 4:**
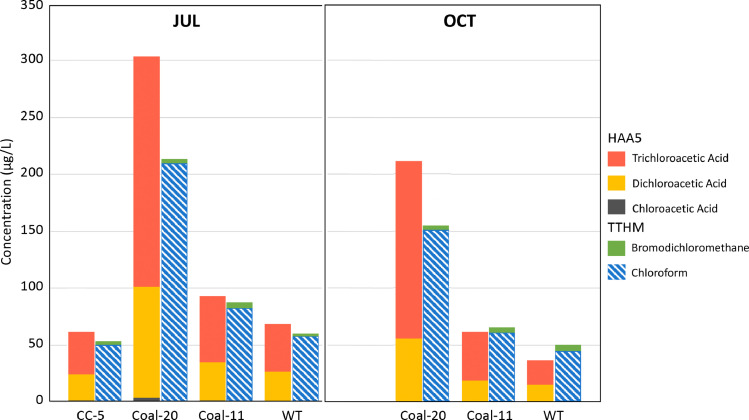
Major DBP group comparisons between formation potential samples and values reported at the water treatment facility in association with actual disinfection for drinking water. Comparisons were made across the 2020 sampling locations for July and October between DBP-FP for creek samples versus actual DBP concentrations reported from the water treatment (WT) facility.

By design, DBP formation potential (DBP-FP) determines all reactive components within a sample. This complete reaction differs from water treatment practice where facilities typically dose disinfectant to achieve two-log inactivation of pathogens likely resulting in lower DBP concentrations. While limited to HAA5 and TTHM, the comparison of quarterly drinking water reports for summer and fall 2020 with temporally aligned sample collections reacted for DBP-FP revealed similar types and fractions of this subset. This was characterized by a dominance of the THMs chloroform and bromodichloromethane and the HAAs dichloroacetic acid and trichloroacetic acid (Fig. 4). The suite of additional DBP-FP compounds analyzed were either not detected or comprised 1% or less of the total DBP concentrations measured with the exception of chloral hydrate in the haloacetaldehydes (HALs) class detected at an average of 5–9% of the total (Fig. S2B). While HALs are not regulated and could not be compared to the historical data, correlative analyses were conducted and revealed analogous correlations with DOC and UV_254_ (Fig. S6). Although unregulated DBP classes (e.g. haloacetaldehydes and haloacetonitriles) occur at lower concentrations than regulated THMs and HAAs, they can contribute more to the cytotoxicity associated with disinfection byproducts than regulated THMs and HAAs in conventional drinking waters (Lau et al., 2020). This lends further credence to the application of UV_254_ screening during synoptic watershed sampling to understand potential DBP formation during drinking water treatment. DBP-FP concentrations can be found in Table S5 and the research data.

### 3.5 Reactivity of DOM functional groups during disinfection

**Fig. 5:**
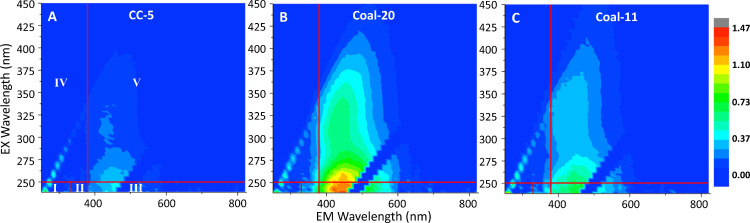
Excitation-emission matrix spectra reveal a hotspot for peak terrestrial organic matter with a fulvic acid-like signature. Spatial differences in July 2020 EEM spectra that are (A) low upstream of the hotspot, (B) exhibit a pronounced increase in fulvic acid-like signatures near the hotspot, and then (C) decrease downstream near the water treatment diversion. The five regions are divided (I-V) per Chen et al., 2003 as (I) Aromatic Protein, (II) Aromatic Protein II, (III) Fulvic acid-like, (IV) Soluble microbial by-product-like, and (V) Humic acid-like. Analogous results were recorded in 2021.

A suite of spectroscopic and spectrometric tools was used to understand the reactive components of Coal Creek water leading to DBP formation. UV_254_ measurements reflected significant correlations that served as a proxy for DBP formation in Coal Creek. Further, EEM spectra indicated the organic compounds present in the creek are mainly sourced from fulvic acid-like organic inputs with a prominent peak associated with terrestrial sourced OM (excitation: 240–250 nm, emission: 381–580 nm) (Chen et al., 2003; Xue et al., 2017). This peak was greatest in the identified hot spot region with reduced peaks downstream (Fig. 5). While spectra were observed in the humic-acid-like region, the humification index did not correlate with HAA5 and TTHM formation potential (*P* = 0.5, 0.4) in contrast to the fluorescence index, (*P* = 0.03, 0.01) (Fig. S6). These correlations agree with the peak analysis in which the fluorescence index has been used to characterize aqueous fulvic acid sources (McKnight et al., 2001).

**Fig. 6:**
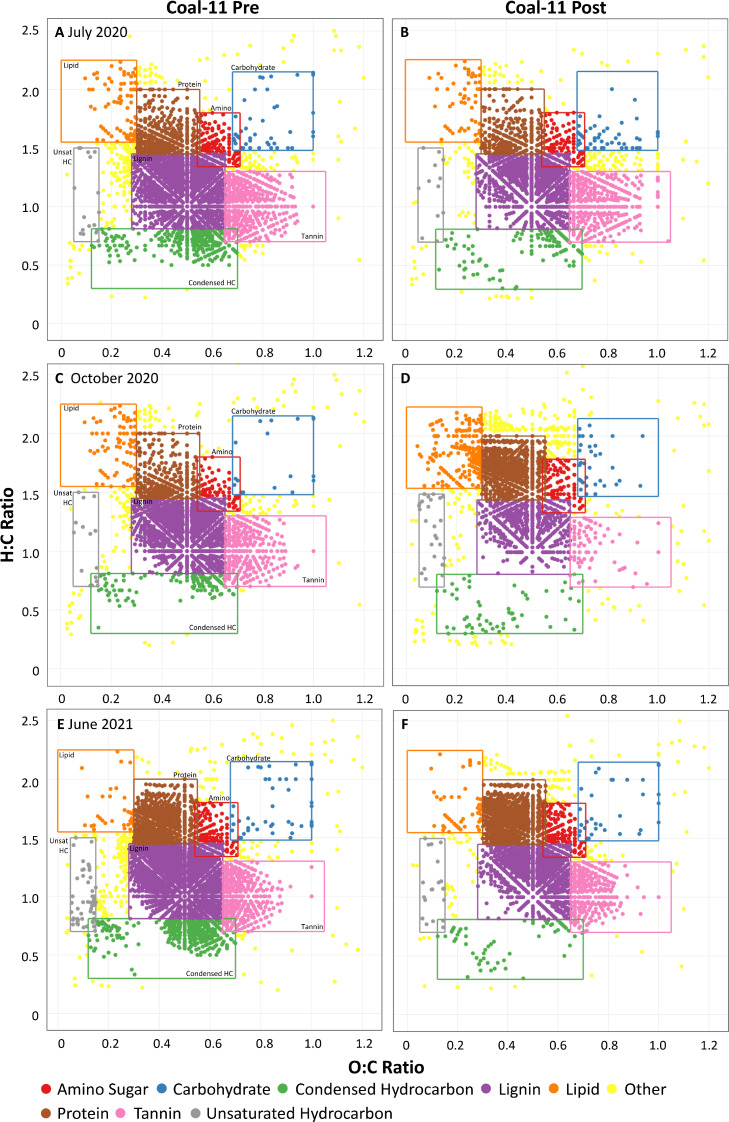
Van Krevelen Diagrams depicting the assignment of chemical classes using O:C and H:C ratios for Coal-11 water samples. Pre-chlorination (Pre) and post-chlorination (Post) relative counts are shown for (A, B) July 2020 samples, (C, D) October 2020 samples, and (E, F) June 2021 samples.

FTICR-MS results of pre and post-hypochlorite-dosed water provided additional insight into the reactive chemical classes within Coal Creek before and after the reaction with chlorine. While not quantitative, the analysis of FTICR data from spatial and temporal samples together was used to statistically identify changes in OM chemical classes associated with chlorination. The chemical classes present in pre-and post-chlorinated samples revealed that the relative counts of lignin-like, tannin-like, and condensed hydrocarbon-like classes decreased after reaction with hypochlorite as highlighted in Van Krevelen diagrams (Figs. 6 and S7).

**Fig. 7:**
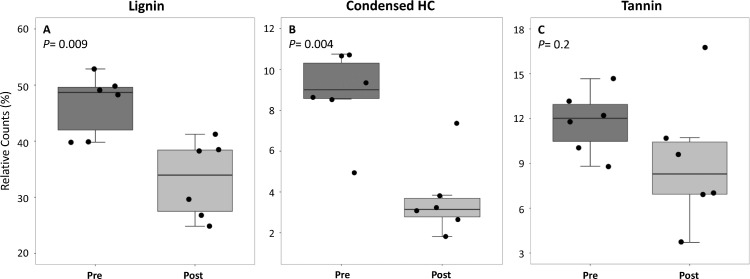
Pre and Post chlorinated samples reveal reactive terrestrial organic matter inputs. Comparisons of pre-chlorinated (Pre) and post-chlorinated (Post) samples reveal changes in relative counts of (A) lignin-like, (B) condensed hydrocarbon-like, and (C) tannin-like, classes. The error bars indicate plus or minus one standard deviation (*n* = 6) for all FTICR-MS samples (Table S2). *P* values are from Mann-Whitney Wilcoxon tests.

Lignin-like molecules comprised the largest relative counts of chemical classes in Coal Creek samples before chlorination and significantly decreased (*P* = 0.009) by 28 ± 13% after chlorination. Significant decreases (55 ± 25%) were also observed for condensed hydrocarbon (HC)-like molecules after chlorination (*P* = 0.004) (Fig. 7). A similar trend, where tannin-like relative counts also decreased, lacked significance. Other analyzed classes (amino-sugar-like, protein-like, etc.) either did not significantly change or increased in relative counts with the addition of chlorine in contrast to the pre-chlorination samples (Fig. S8).

The reactive chemical classes reduced in average relative counts after chlorination have been linked to the formation of DBP compounds previously. Specifically, higher trichloroacetic acid formation has been associated with lignin phenols while chloroform with humic and fulvic acids (Hua et al., 2014). Further, lignin phenols have been highlighted as important precursors to the unregulated class of haloacetaldehydes (Chuang et al., 2015). A more recent study further corroborates these findings from drinking source waters in Spain in which lignin was deemed a major component of natural surface waters and most reactive after chlorination along with condensed hydrocarbons. The complexity of these reactions was also highlighted, as the authors observed increases in certain lignin signals (Sanchís et al., 2020). Further, given the presence of lignin content in evergreen needles (Leonard et al., 2020), the spruce forest landcover could be a contributing source of lignin from the Ohio Fork. While not investigated further herein, this or other landcover contributions within the identified domain could provide clues toward reactive organic matter sources in similar watersheds.

## 4 Conclusions and implications

We present an approach that combines historical DBP drinking water quality archives, synoptic sampling, and targeted spectroscopic characterizations of pre and post chlorinated creek samples to better understand a temporally and spatially dynamic watershed that harbors a pronounced OM export control point. Hydrographs were used to inform synoptic sampling and identify a hot spot region in Coal Creek that exhibits seasonal, temporal, and spatial trends for increased OM export. Targeted spectroscopic analyses contrasting chlorine-reacted and unreacted waters revealed that lignin-like and condensed hydrocarbon-like classes reacted most strongly. These classes likely contributed to heightened DBP formation during drinking water treatment and provide potential clues to identify watershed sources of reactive organic matter that may inform subsequent hydrobiogeochemical and drinking water treatment studies.

This study highlights an opportunity for more effective water management coupled with treatment that includes comprehensive monitoring plans from the source to tap. Due to the repeatability of the hot moments associated with seasonal hydrologic processes, recurrent high DBP concentrations can be proactively addressed and peaks more precisely identified. Should these variables shift with climate change as suggested by our limited 15 year data set, certain hot moments could become “hotter” from increased organic release to surface waters during climate-induced stress (McDaniel et al., 2014), suggesting this type of analysis could serve as a predictive tool to mitigate risk. Possible solutions include alternative disinfection methods such as UV irradiation and more aggressive TOC attenuation through processes such as coagulation, activated carbon, biofiltration, or nanofiltration. Alternatively, DBPs can be removed after formation with membrane filtration, air stripping, or granular activated carbon (Zazouli and Kalankesh, 2017); such technologies could be implemented during periods with the highest risk for formation. However, solutions beyond treatment should also be explored as these technological upgrades are not always economical for smaller treatment facilities such as Crested Butte. In this case, a more precise understanding of the temporal and spatial drivers of export peaks could be integrated into solutions such as temporary sourcing from a secondary water supply such as Wildcat Creek during the peak discharge window. Our findings more broadly reveal how seasonal and climate-induced changes in hydrobiogeochemical processes have implications for human health and well-being, providing an opportunity for a proactive and holistic strategy that integrates drinking water treatment and watershed management to address this challenge.

## Research data

The raw water quality datasets are available in the Supplementary Information (SI). Raw data not included in the SI is available at GitHub: https://github.com/ltleonard/Leonard-et-al.-DBP. Data sourced from other entities are cited as such in the Methods & Materials.

## Declaration of Competing Interest

The authors declare that they have no known competing financial interests or personal relationships that could have appeared to influence the work reported in this paper.

## Appendix Supplementary materials

Image, application 1
