# Supplementary material for: Disinfection byproducts formed during drinking water treatment reveal an export control point for dissolved organic matter in a subalpine headwater stream
Source: Water Res X. 2022 Apr 25;15:100144. doi: 10.1016/j.wroa.2022.100144 (PMC9079345; doi:10.1016/j.wroa.2022.100144)
Supplement: Supplementary file 1 [file mmc1.pdf]

Supporting Information for:

Disinfection byproducts formed during drinking water treatment reveal an export control point for dissolved organic matter in a subalpine headwater stream.

Submitted to Water Research

Laura T. Leonard[1], Gary F. Vanzin[1], Vanessa A. Garayburu-Caruso[2], Stephanie S. Lau[3], Curtis A. Beutler[4], Alexander W. Newman[4], William A. Mitch[3], James C. Stegen[2], Kenneth H. Williams[4,5], Jonathan O. Sharp[1, 6*]

[1]Department of Civil and Environmental Engineering, and [6]Hydrologic Science and Engineering Program, Colorado School of Mines, Golden, CO 80401, USA
[2]Pacific Northwest National Laboratory, Richland, WA 99354
[3]Stanford University, Stanford, CA 94305
[4]Rocky Mountain Biological Laboratory, Gothic, CO, 81224, USA
[5]Lawrence Berkeley National Laboratory, Berkeley, CA, 94720, USA
[*]Corresponding author: Jonathan O. Sharp, Email address: jsharp@mines.edu

This supporting information includes 12 pages, 5 tables, and 8 figures.

List of Tables

Table S1 ____ 2
Table S2 ____ 3
Table S3 ____ 7
Table S4 ____ 8
Table S5 ____ 10

List of Figures

Figure S1 ____ 2
Figure S2 ____ 4
Figure S3 ____ 5
Figure S4 ____ 5
Figure S5 ____ 6
Figure S6 ____ 9
Figure S7 ____ 11
Figure S8 ____ 12

Table S1. Snow telemetry (SNOTEL) climate summaries for Mt. Crested Butte, elevation 3,100 m.

Water Year (Oct-Sep)	Dry Soil[1]	Summer Days[2]	Ice Days[3]	Frost Days[4]	Total Precipitation (cm SWE)	Snow (cm SWE)
2015	0	49	52	183	79	38
2016	39	60	66	196	82	43
2017	143	73	59	171	94	61
2018	163	88	38	174	48	31
2019	44	69	73	205	88	63
2020	286	83	71	203	62	39
**AVG (1990-2010)**	**132 ± 90**	**70 ± 27**	**75 ± 29**	**206 ± 31**	**80 ± 15**	**48 ± 14**

[1]Total days max soil water % < 10 at 20 cm depth. Data was available starting in 2006, thus the average is 2006-2016.
[2]Total days with TMax > 20 ° C
[3]Total days with TMax < 0 ° C
[4]Total days with TMin < 0 ° C

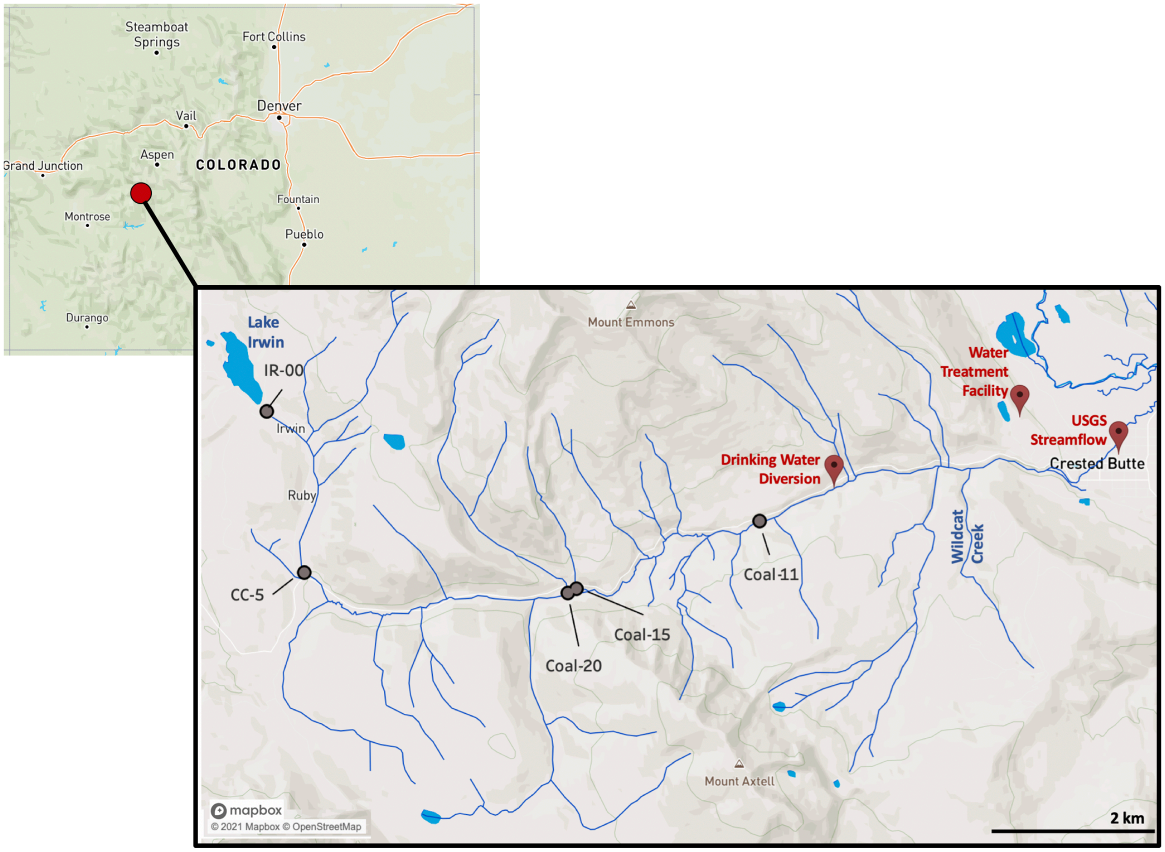

Figure S1. Coal Creek transect. The select 2020 sampling locations are shown in grey circles. Also shown is the drinking water diversion. Coal-11 is located upstream of the diversion where historical DOC measurements were collected. Also shown is the water treatment facility that reported DBP concentrations and the USGS streamflow monitoring location. The base map was created in Mapbox with points plotted in Tableau.

Table S2. Summary of water analyses conducted during 2020 and 2021 sample collections.

	UV_{254}	DOC	EEM Spectra	DBP-FP	FTICR-MS
**Volume of sample**	1 mL	20 mL	1 mL	2x 250 mL	60 mL
**Chlorination**	Pre	Pre	Pre	Post	Pre and Post
**Samples Analyzed***	All Samples	All 2020 samples, and select 2021 samples in Tables S4, S5	July 2020 CC-5, Coal-11, Coal-20	July 2020 CC-5, Coal-11, Coal-20	July 2020 Coal-11
			-	Oct 2020 Coal-11, Coal-20	Oct 2020 Coal-11, Coal-15, Coal-20
			June 2021 Ohio, CC-7, Coal-11, Coal-20	June 2021 Coal-11, Coal-20	June 2021 Coal-11, Coal-20

*July 30, 2020: CC-5, Coal-11, Coal-20
October 9, 2020: Coal-11, Coal-15, Coal-20
June 8, 2021: Synoptic Coal Creek transect
July 28, 2021: Synoptic Coal Creek with Ohio transect

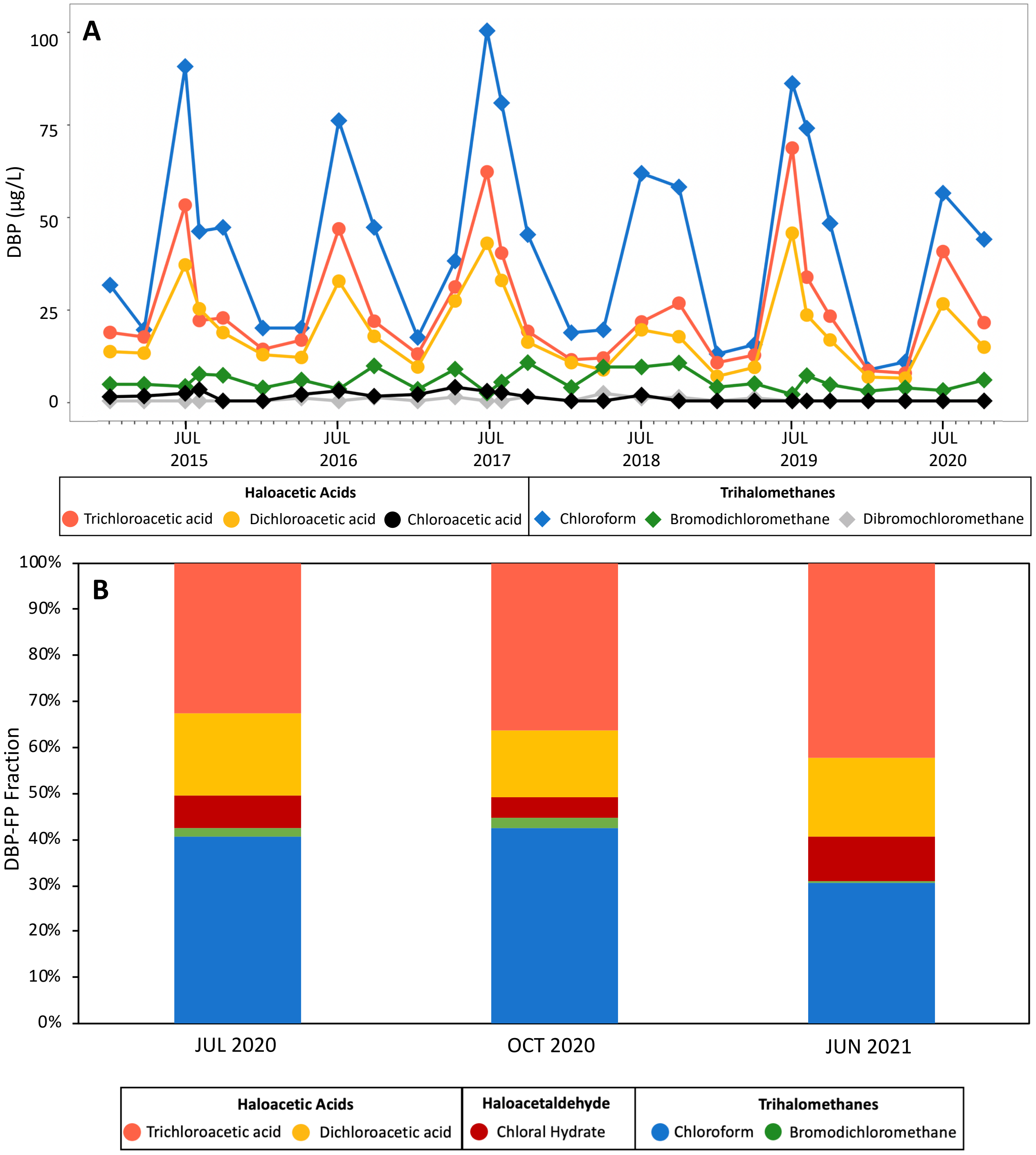

Figure S2. Major DBP compounds measured in the historical and FP datasets. (A) DBP concentrations and associated fractions of the regulated HAA5 and TTHM groups present in the historical data, and (B) the fraction of the total DBP-FP concentrations detected that comprised more than 1% of the average across all samples for each sample date.

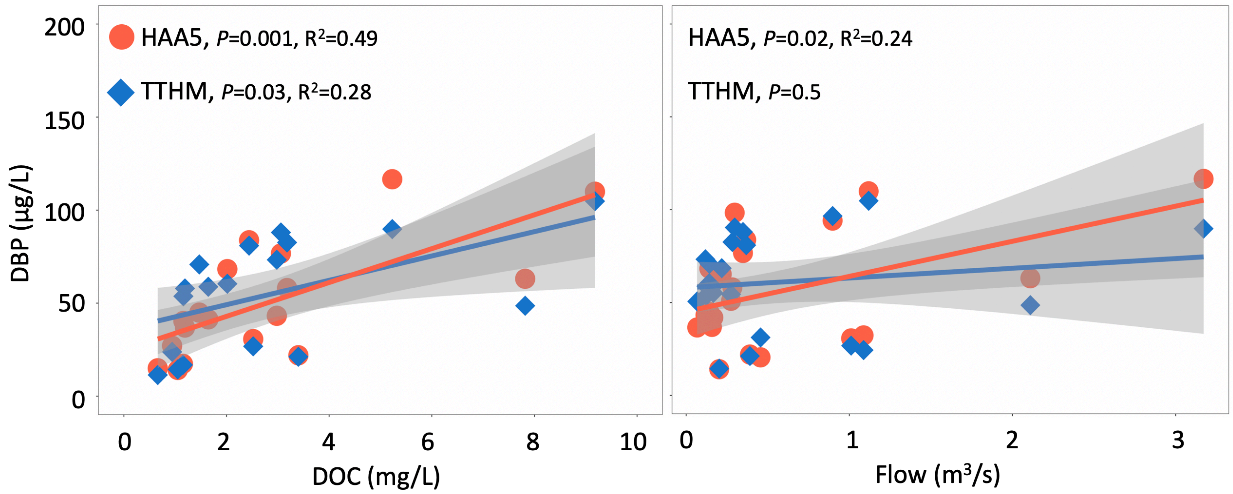

Figure S3. Correlation plots between peak DBP concentrations and the same-day flow and DOC concentrations. Values represent the five-year daily Coal Creek DOC concentrations and flow from the USGS hydrograph associated with the quarterly reported DBP concentrations from the treatment facility. Confidence intervals of 0.95 are shown in grey shading for each fit.

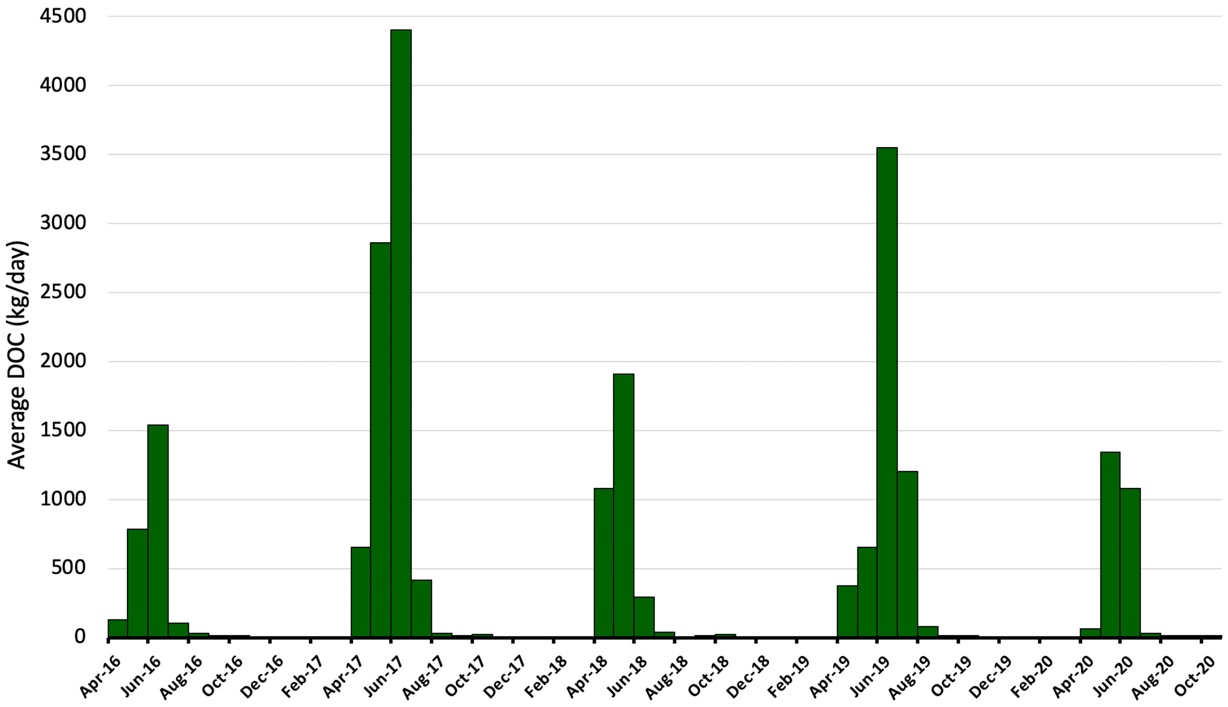

Figure S4. Average monthly DOC loading per year.

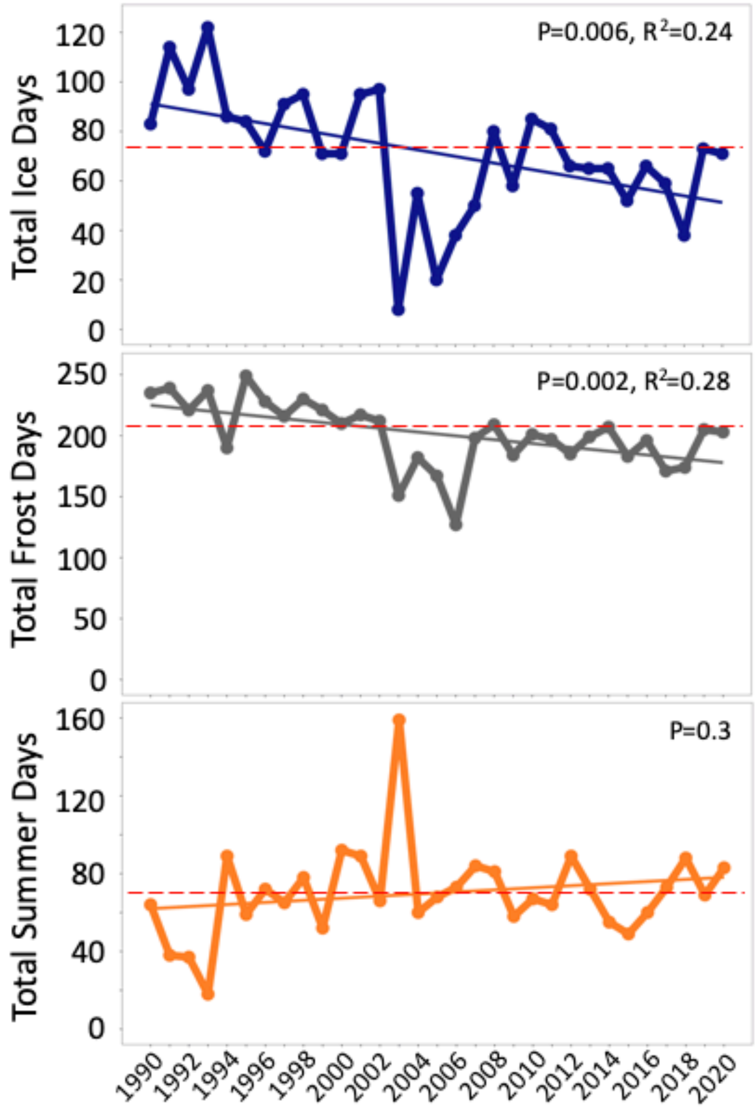

Figure S5. Climate trends in Crested Butte for total ice, frost, and summer days. The 1990-2010 normal value is shown in the dotted red line. Linear trendlines and the respective *P* values indicate linear fit significance. R^2 values are shown only for significant trends. Total ice days represent the total days the daily maximum was less than 0° C, total frost days are total days the daily minimum was less than 0° C, and summer days are total days the maximum temperature was greater than 20° C.

Table S3. UV_{254} and DOC results from the June 2021 synoptic sampling with estimated DBP values based on linear correlation equations. Coal-20 and Coal-11 were tested for actual DBP-FP concentrations to compare to the estimated values and are shown in bold.

Sample	UV_{254} (abs)	Estimated HAA5 (µg/L)	Estimated TTHM (µg/L)	DOC (mg/L)	Estimated HAA5 (µg/L)	Estimated TTHM (µg/L)
IR-00	0.08	212	144	2.5	242	158
CC-2	0.17	485	268	4.7	542	293
CC-3	0.16	467	260	4.5	508	278
Coal-30	0.17	496	273	4.7	538	292
CC-5	0.17	512	280	4.9	557	300
Ohio	0.34	1024	511	8.8	1076	534
CC-6	0.24	703	366	5.7	673	352
CC-7	0.34	1005	503	8.4	1025	511
CC-8	0.27	817	418	7.0	836	426
Splains	0.23	679	356	-	-	-
CC-9	0.25	753	389	6.6	784	403
Elk-00	0.10	282	176	-	-	-
Elk-East	0.04	98	93	-	-	-
**Coal-20**	**0.26**	**762**	**393**	**6.5**	**770**	**396**
Coal-15	0.23	667	350	5.7	663	348
CC-10	0.22	642	339	5.5	647	341
Evans	0.04	92	90	-	-	-
CC-11	0.19	563	303	5.2	604	321
Coal-12	0.20	594	317	5.4	636	335
BOG-00	0.03	54	73	-	-	-
BOG-02	0.02	45	69	-	-	-
BOG-03	0.03	74	82	-	-	-
BOG-04	0.04	106	97	-	-	-
CC-11.5	0.20	587	314	5.2	608	323
**Coal-11**	**0.19**	**552**	**298**	**4.9**	**561**	**302**
Keystone	0.02	42	68	-	-	-
Coal-5	0.17	499	274	4.6	529	287
Red Lady	0.02	30	62	-	-	-
Wild-00	0.11	311	189	3.3	352	208
CC-12	0.16	453	254	4.4	495	272
Coal-2	0.16	470	261	4.5	513	280

Table S4. UV_{254} and DOC results from the July 2021 synoptic sampling with estimated DBP values based on linear correlation equations. Coal-20 and Coal-11 are shown in bold.

Sample	UV_{254} (abs)	Estimated HAA5 (µg/L)	Estimated TTHM (µg/L)	DOC (mg/L)	Estimated HAA5 (µg/L)	Estimated TTHM (µg/L)
IR-00	0.06	158	120	2.1	192	135
CC-2	0.08	229	152	-	-	-
CC-3	0.07	179	130	-	-	-
Coal-30	0.09	240	157	-	-	-
CC-5	0.10	269	171	-	-	-
Ohio Creek	0.14	412	235	3.8	413	235
Ohio-2	0.17	482	267	4.1	459	256
Ohio-3	0.17	499	274	5.0	571	306
Ohio-4	0.17	490	270	5.0	578	310
Ohio Pass	0.10	272	172	-	-	-
CC-6	0.17	486	269	4.4	502	275
CC-7	0.16	479	265	-	-	-
SPG3	0.28	824	421	7.1	860	437
CC-8	0.16	454	254	4.3	483	267
Splains	0.06	168	125	-	-	-
CC-9	0.11	321	194	-	-	-
Elk-00	0.02	30	63	-	-	-
Elk-East	0.02	38	66	-	-	-
**Coal-20**	**0.11**	**309**	**189**	**3.4**	**366**	**214**
Coal-15	0.08	209	143	-	-	-
CC-10	0.06	160	121	-	-	-
Evans	0.02	52	72	-	-	-
CC-11	0.06	173	127	-	-	-
Coal-12	0.06	167	124	-	-	-
BOG-00	0.02	23	59	-	-	-
BOG-02	0.01	15	56	-	-	-
BOG-03	0.02	52	73	-	-	-
BOG-04	0.04	84	87	-	-	-
CC-11.5	0.06	154	118	-	-	-
**Coal-11**	**0.06**	**150**	**117**	**2.1**	**185**	**132**
Keystone	0.00	-	-	-	-	-
Coal-5	0.03	69	80	-	-	-
Red Lady	0.02	27	61	-	-	-
Wild-00	0.06	146	115	-	-	-
CC-12	0.04	108	97	-	-	-

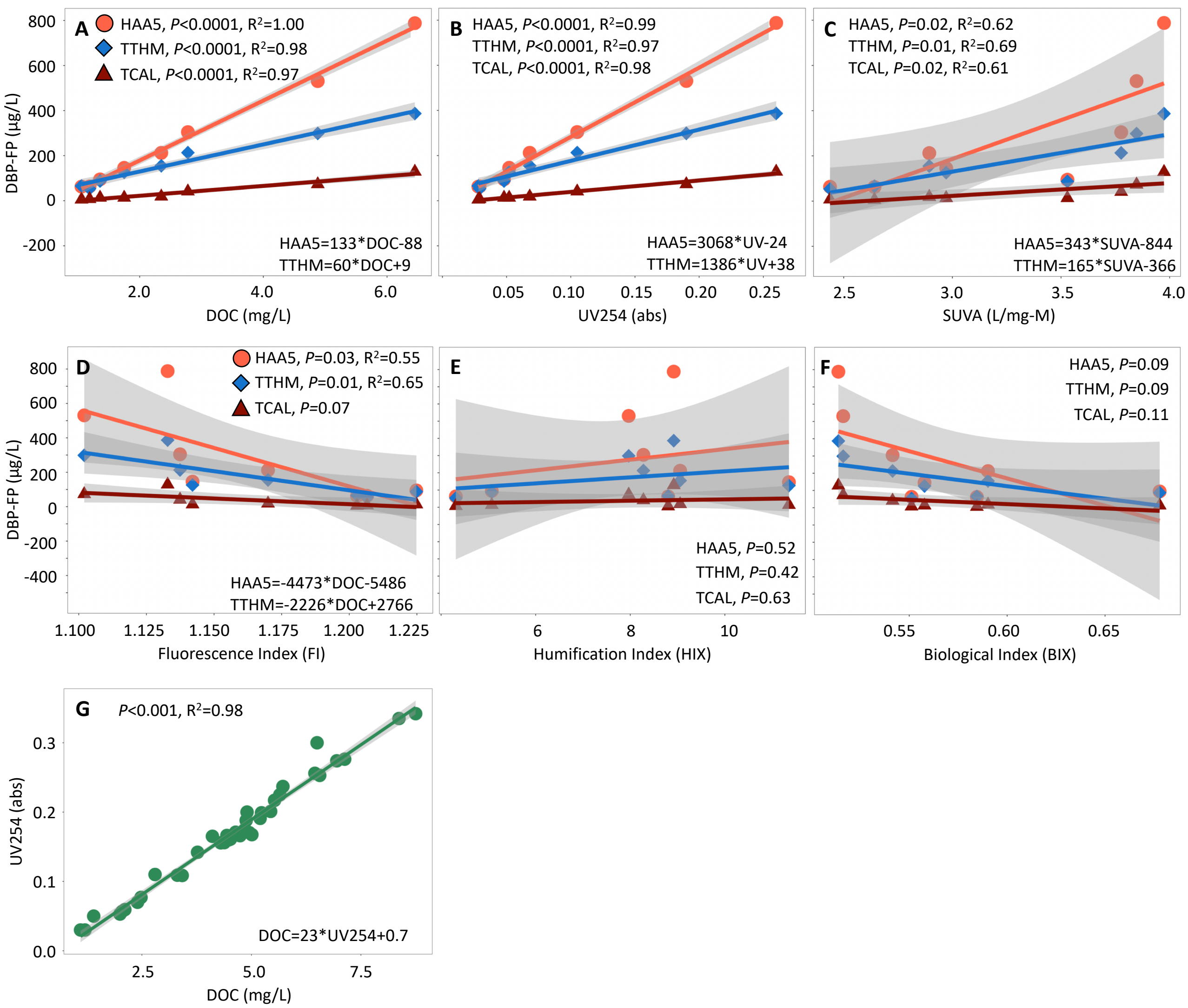

Figure S6. Correlation plots of (A) DOC, (B) UV_{254}, (C) SUVA, (D) Fi, (E) Hix, and (F) Bix between TTHM and HAA5 and TCAL (Chloral Hydrate). In addition, a correlation plot of (G) DOC and UV_{254} from the 2021 synoptic samples are shown. All DBP concentrations are based on creek DBP formation potential tests. Confidence intervals of 0.95 are shown in grey shading. Linear trendlines and the respective *P* values indicate linear fit significance. R^2 values are shown only for significant trends.

Table S5. Detailed analysis of reactive components and their DBP formation potential for the select samples along the creek transect.

Sample	Date	DOC (mg/L)	UV_{254} (abs)	SUVA (L/mg-M)	TTHM (µg/L)	HAA5 (µg/L)	TCAL (µg/L)	Hix	Fi	Bix	Cond HC (%)*	Tannin (%)*	Lignin (%)*
**CC-5**	7/30/20	1.2	0.03	2.4	53	62	7	4.3	1.2	0.6	-	-	-
**Coal-20**	7/30/20	2.8	0.11	3.8	213	303	41	8.3	1.1	0.5	-	-	-
**Coal-11**	7/30/20	1.4	0.05	3.5	87	93	13	5.1	1.2	0.7	9	12	48
**Coal-20**	10/9/20	2.4	0.07	2.9	155	211	19	9.1	1.2	0.6	11	13	40
**Coal-15**	10/9/20	1.8	0.05	3.0	126	145	14	11.4	1.1	0.6	11	15	40
**Coal-11**	10/9/20	1.1	0.03	2.6	66	61	6	8.8	1.2	0.6	5	12	53
**Coal-20**	6/8/21	6.5	0.26	4.0	387	787	129	8.9	1.1	0.5	9	10	49
**Coal-11**	6/8/21	4.9	0.19	3.8	298	530	73	8.0	1.1	0.5	9	9	50

*Relative counts of chemical classes

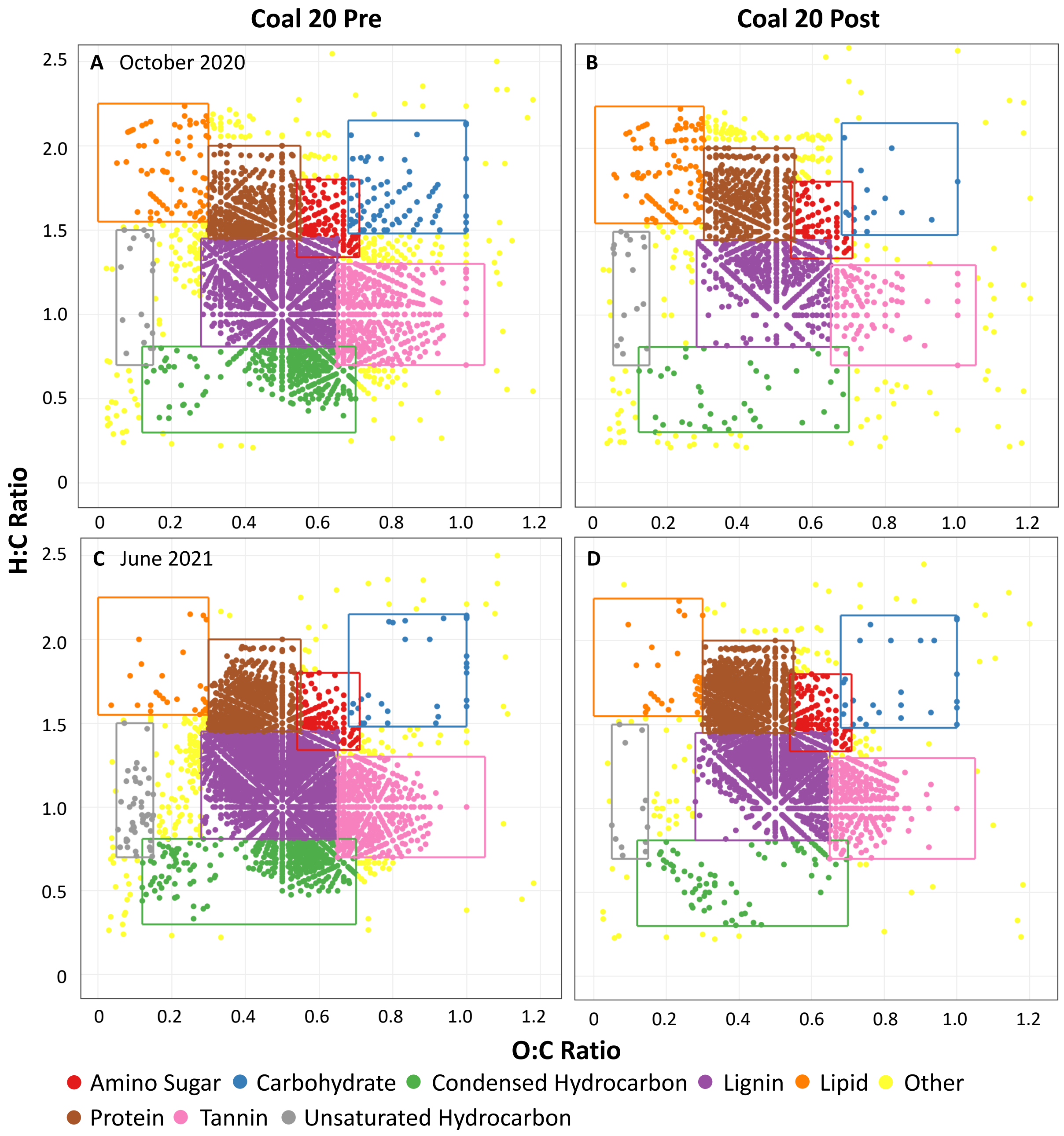

Figure S7. Van Krevelen Diagrams for Coal 20 samples. Pre-chlorination (Pre) and post-chlorination (Post) relative counts are shown for (A, B) October 2020 samples, and (C, D) June 2021 samples.

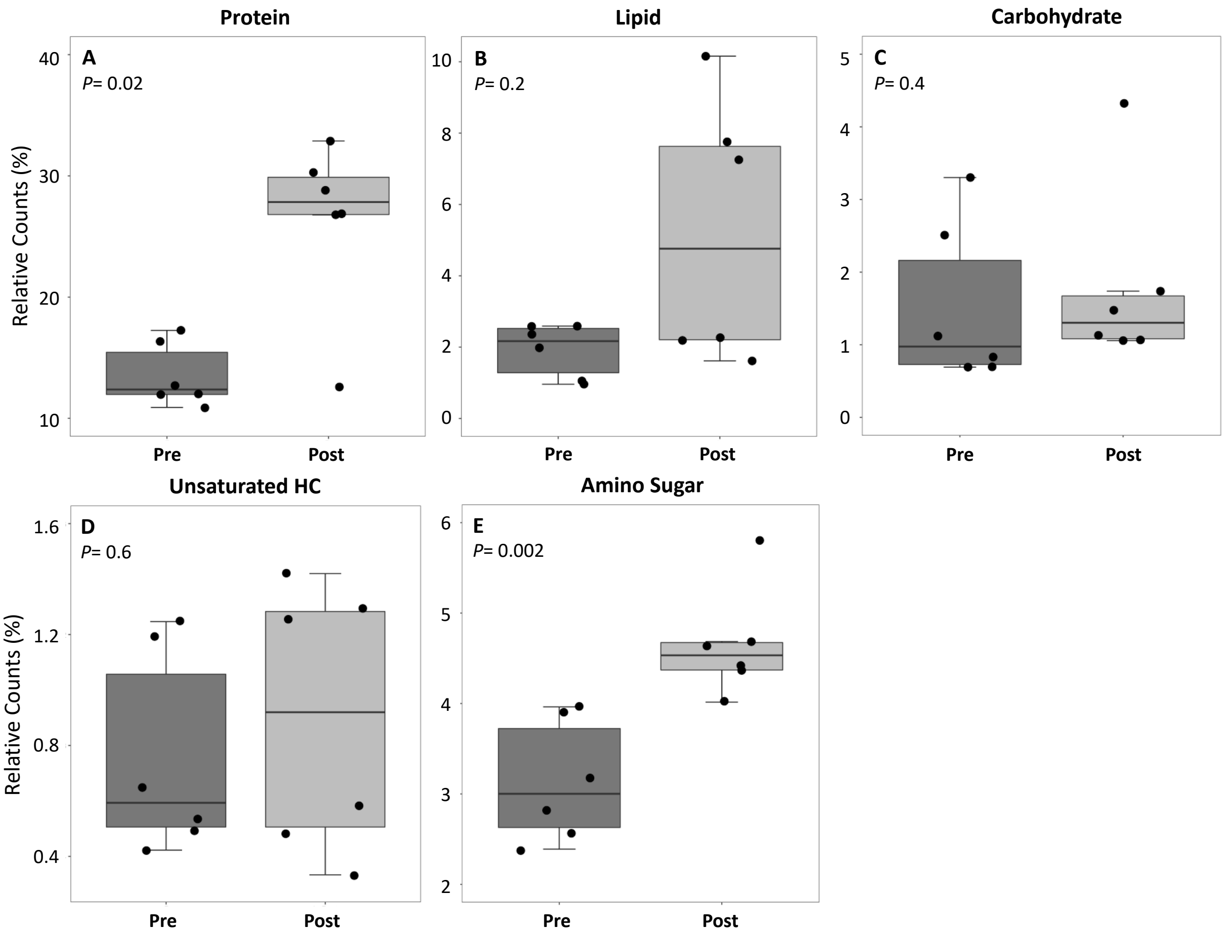

Figure S8. Average relative abundances of the other analyzed organic classes. Pre-chlorination (Pre) and post-chlorination (Post) relative counts are shown based on the average of all samples collected in 2020 and 2021 for the other chemical classes analyzed with FTICR-MS. The error bars indicate plus or minus one standard deviation (n=6) for all FTICR-MS samples (Table S2). *P* values are from Mann-Whitney Wilcoxon tests.
